# Changes to the Human Serum Proteome in Response to High Intensity Interval Exercise: A Sequential Top-Down Proteomic Analysis

**DOI:** 10.3389/fphys.2019.00362

**Published:** 2019-04-02

**Authors:** Nigel Kurgan, Nour Noaman, Melissa R. Pergande, Stephanie M. Cologna, Jens R. Coorssen, Panagiota Klentrou

**Affiliations:** ^1^Department of Kinesiology, Brock University, St. Catharines, ON, Canada; ^2^Centre for Bone and Muscle Health, Brock University, St. Catharines, ON, Canada; ^3^Department of Health Sciences, Brock University, St. Catharines, ON, Canada; ^4^Department of Biological Sciences, Brock University, St. Catharines, ON, Canada; ^5^Molecular Medicine Research Group, Department of Molecular Physiology, School of Medicine, Western Sydney University, Campbelltown, NSW, Australia; ^6^Department of Chemistry, University of Illinois at Chicago, Chicago, IL, United States

**Keywords:** exerkines, inflammation, exercise intensity, HIIE, biomarkers, proteoforms, top-down proteomics, two-dimensional gel electrophoresis

## Abstract

Exercise has been shown to improve health status and prevent chronic diseases. In contrast, overtraining can lead to maladaptation and detrimental health outcomes. These outcomes appear to be mediated in part by released peptides and, potentially, alterations in protein abundances and their modified forms, termed proteoforms. Proteoform biomarkers that either predict the beneficial effects of exercise or indicate (mal)adaptation are yet to be elucidated. Thus, we assessed the influence of high-intensity interval exercise (HIIE) on the human serum proteome to identify novel exercise-regulated proteoforms. To this end, a top-down proteomics approach was used, whereby two-dimensional gel electrophoresis was used to resolve and differentially profile intact proteoforms, followed by protein identification via liquid chromatography-tandem mass spectrometry. Blood was collected from six young-adult healthy males, pre-exercise and 5 min and 1 h post-exercise. Exercise consisted of a maximal cycle ergometer test followed by 8 min × 1 min high-intensity intervals at 90% *W*_max_, with 1 min non-active recovery between intervals. Twenty resolved serum proteoforms changed significantly in abundance at 5 min and/or 1 h post-HIIE, including apolipoproteins, serpins (protease inhibitors), and immune system proteins, known to have broad anti-inflammatory and antioxidant effects, involvement in lipid clearance, and cardio-/neuro-protective effects. This initial screening for potential biomarkers indicates that a top-down analytical proteomic approach may prove useful in further characterizing the response to exercise and in understanding the molecular mechanisms that lead to health benefits, as well as identifying novel biomarkers for exercise (mal)adaptation.

## Introduction

It is well-documented that regular exercise is associated with numerous health benefits (Smorawinski et al., 2000; Warburton et al., 2006). Mechanisms that lead to the numerous health benefits associated with exercise training may be stimulated through multiple bouts of acute (single-session) exercise, i.e., high-intensity interval exercise (HIIE), that occurs over a time-period of training (Hojman et al., 2018). Specifically, it is hypothesized that HIIE elicits alterations to protein function or content in blood, contributing to the numerous multi-system health benefits observed with continued training. However, excessive training can also lead to an apparent catabolic and systemic inflammatory state (i.e., mal-adaptation) (Kurgan et al., 2018).

In order to better elucidate mechanisms underlying beneficial and/or detrimental effects of exercise, alterations to the skeletal muscle proteome (Petriz et al., 2017) [which may influence/be influenced by the plasma/serum proteome(s)], plasma peptidome (Parker et al., 2017; Santos-Parker et al., 2018), serum metabolome (Nieman et al., 2013; Pechlivanis et al., 2013), and plasma extracellular vesicle content (Whitham et al., 2018) have been investigated. Recently, a cross-sectional assessment of the effects of high and low levels of physical activity on the plasma proteome utilizing an aptamer-based SOMAscan proteomic assay has also been reported (Santos-Parker et al., 2018). Collectively, these have found large perturbations in bioactive peptides, metabolic pathways (e.g., glycolysis), cell cycle regulatory proteins, and proteins related to immunity. However, untargeted and comprehensive assessments of plasma and/or serum proteomes in response to HIIE – inclusive of gene, splice, and post-translationally modified variants (i.e., proteoforms), which are influenced by metabolic needs and health/disease state (Jungblut et al., 2008; Thiede et al., 2013; Coorssen and Yergey, 2015) – are yet to be carried out.

Exercise-regulated changes in the abundance of targeted proteins within serum have been found to profoundly effect cell proliferation and differentiation *in vitro* (e.g., anti-cancer effects) (Hojman et al., 2018). Increased serum collagen leading to increased tensile strength in engineered ligaments has also been observed (West et al., 2015). Many of the observed health benefits to exercise have been attributed to a select number of myokines, including IL-6 (Pedersen, 2012), IGF-1 (Hamrick et al., 2010), BDNF (Pedersen et al., 2009), and irisin (Bostrom et al., 2012; Pedersen and Febbraio, 2012). Alterations with positive effects on the immune response, including increased plasma and serum IL-6 and natural killer cell (NK) tumor infiltration (Pedersen et al., 2016), as well as inhibition of toll-like receptors (TLR), recruitment of M1 macrophages and CD8^+^ T lymphocytes (reviewed in: Lancaster and Febbraio, 2014), and NK gene expression and microRNA changes associated with cancer and cell communication (e.g., p53 signaling pathway) (Radom-Aizik et al., 2013), are also reported. Thus, here we assessed the immediate and delayed impacts of HIIE on the human serum proteome to further elucidate underlying molecular mechanisms and identify novel candidate biomarkers (e.g., exercise regulated factors/exerkines) for the therapeutic and disease-preventative effects of exercise.

To assess intact proteoforms rather than simply amino acid sequences, two-dimensional gel electrophoresis (2DE), coupled with liquid chromatography-tandem mass spectrometry (LC-MS/MS) for subsequent protein identification, was utilized to differentially profile the proteome of whole serum, preceding, immediately following, and an hour following HIIE. This top-down approach, which currently offers the most reproducible, comprehensive resolution and quantitative detection of intact proteoforms (Oliveira et al., 2014; Coorssen and Yergey, 2015; Zhan et al., 2018), revealed several changes following HIIE which may, with further investigation, improve our understanding of and predictions for health outcomes of exercise.

## Materials and Methods

### Materials

Where applicable, consumables were of electrophoresis grade or higher. Vacutainer^®^ SST (serum-separator tubes) and 21G butterfly needles were from BD (Franklin Lakes, NJ, United States). ReadyStrip^TM^ immobilized pH gradient (IPG) strips (17 cm, pH 3–10 non-linear), Bio-Lyte carrier ampholytes (pH 3-10, pH 4-6), and 2-D SDS-PAGE Standards were from Bio-Rad Laboratories (Hercules, CA, United States). AEBSF, agarose I, bovine serum albumin (BSA), CHAPS, dithiothreitol (DTT), leupeptin, mineral oil, and TG-SDS buffer concentrate were from Amresco (Solon, OH, United States). Acetic acid was from Anachemia (Montreal, Quebec); sodium dodecyl sulfate (SDS) was from J. T. Baker Chemical Co. (Phillipsburg, NJ, United States); mass spectrometry-grade trypsin was from G-Biosciences (St. Louis, MO, United States); Coomassie Brilliant Blue G-250 (CBB) was from Genlantis (San Diego, CA, United States); and Broad-range (200–10 kDa) Unstained Protein Standard was from New England Biolabs (Ipswich, MA, United States). Ammonium persulfate and aprotinin were from Thermo Fisher Scientific (Waltham, MA, United States). Acetonitrile, formic acid, and methanol were from EMD Millipore (Burlington, MA, United States). Acrylamide/bis-acrylamide (37.5:1) solution and all other chemicals utilized were from Alfa Aesar (Haverhill, MA, United States). Double glass-distilled water (ddH_2_O) was used throughout.

### Study Design, Body Composition and VO_2max_ Measurements

**FIGURE 1:**
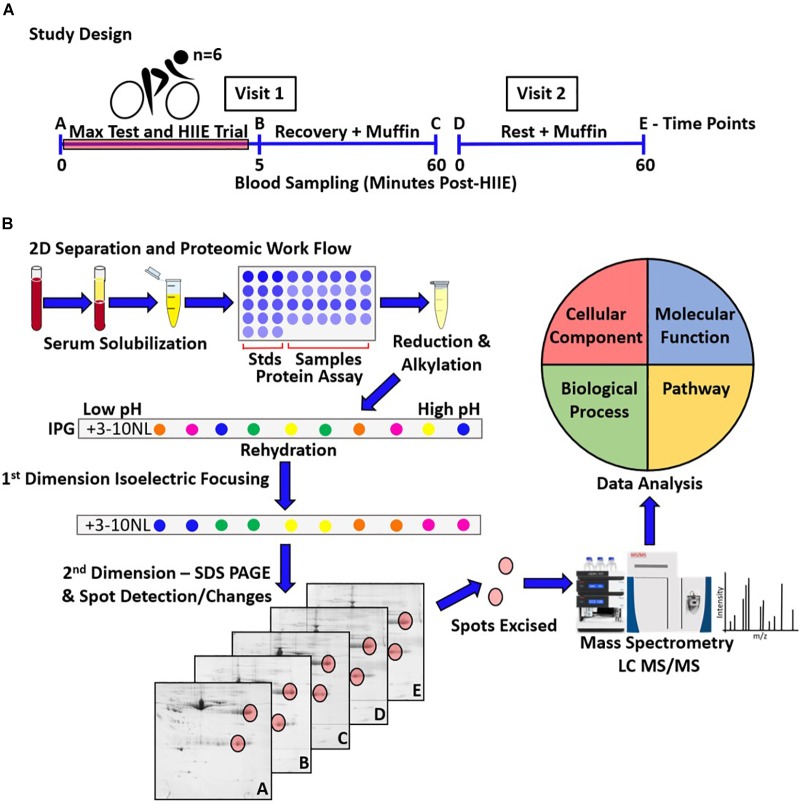
Schematic representation of the study design, and proteomic work flow (i.e., 2DE-LC-MS/MS) for the assessment of the serum proteome following HIIE and during recovery. **(A)** The studied sample consisted of 6 healthy young adult males, who performed one acute bout of HIIE. Blood sampling occurred on an exercise trial visit, at pre- **(A)**, and 5 min **(B)** and 1 h post-HIIE **(C)** (visit 1), as well as on a control trial visit, pre- **(D)** and post-muffin **(E)** (visit 2). **(B)** Triplicates for samples taken at the 5-time points for each participant were resolved by 2DE (90 gels in total) and used to assess spot changes over time. LC-MS/MS was used to analyze and identify the proteoforms present within the protein spots of interest, and gene ontology (Uniprot) and PANTHER were used to analyze and catalog the functions/interactions and biological pathways potentially involved.

This study was approved by the Brock University ethics committee and was conducted in accordance with the Declaration of Helsinki II. Written informed consent was obtained from each participant prior to beginning the study. Study design is outlined in Figure 1A. Briefly, six healthy male participants [age = 24.5 ± 1.3 years; weight = 85.8 ± 10.3 kg; height = 184.1 ± 5.1 cm; body fat% = 12.8 ± 6.8% (mean ± SD)] volunteered to participate, with testing conducted at the Applied Physiology Lab, Brock University. Height was measured with a stadiometer to the nearest millimeter. Body composition was measured via air displacement plethysmography (BodPod; Life Measurement, Inc., United States) to obtain measures of body, fat, and fat-free mass (kg), and body fat percentage. Using a continuous, incremental exercise protocol (described in more detailed below), VO_2max_ was measured on a cycle ergometer (Monark, Vansbro, Sweden). Heart rate was recorded continuously during the assessment using a chest band heart monitor (TIMEX Group Inc., Toronto, ON, Canada), and metabolic gasses were analyzed using an AEI metabolic cart (Model S-3A, AIE Technologies, Pittsburgh, PA, United States). A respiratory gas exchange ratio of at least 1.15 and a heart rate >90% of age-predicted maximal heart rate were criteria for achieving peak aerobic capacity.

For the HIIE trials, participants were asked to abstain from exercise for 24 h prior and to arrive in a fasted state (at least 12 h). Testing began between 0700 and 0900 h, with one participant tested at a time. Upon arrival, the participant rested in a seated position for 15 min prior to their first rested blood draw (A). Venous blood was collected from the medial cubital vein into a 5 ml clot activator serum collection tube. The participant then warmed up on a cycle ergometer for 2 min at 50 W before undergoing a maximal exercise (VO_2max_) test that started at 50 W and increased by 1.5 W every second. The participant was required to remain seated and maintain ≥80 revolutions per minute (RPM). The test was concluded once the participant either stood up from their seat or fell below 60 RPM. The maximum workload (*W*_max_) was then recorded as the maximum power achieved at the final stage of the incremental test. A 5–10 min recovery period was permitted prior to commencing HIIE, which consisted of 8 min × 1 min trials at 90% *W*_max_, with 1 min non-active recovery between trials. Heart rate was continuously recorded as above. Following the HIIE trials, exertion was measured by the Borg scale (Borg, 1998).

The participant was permitted to cool-down for 2 min at 50 W before stepping off the cycle ergometer for the 2nd blood draw, taken ∼5 min post-HIIE to assess acute effects of exercise (B). Following this blood draw the participant consumed ∼250 ml of water and one store-bought blueberry muffin (∼115 g). Blood was drawn 1 h post-HIIE to assess recovery from exercise (C).

A second visit parallel to the first except *without* exercise, in which blood was drawn prior to (D) and 1 h following the consumption of a blueberry muffin (E), served to control for potential day-to-day variability as well as food consumption.

### Sample Preparation

2D separation and proteomic work flow is outlined in Figure 1B. The initial step of this work flow was sample preparation. Blood samples in collection tubes were inverted 5 times and allowed to clot at room temperature for 25 min prior to 4°C centrifugation at 1,500 × *g* for 15 min. Serum was collected and supplemented with kinase, phosphatase, and protease inhibitors [inhibitor cocktail: 4 μM staurosporine; 5 mM sodium fluoride; 1 mM benzamidine; 0.2 mM DTT; 0.3 μM aprotinin; 0.6 μM pepstatin A; 0.85 μM leupeptin; 8 μM AEBSF (Coorssen et al., 1998; Butt and Coorssen, 2005)]. Serum aliquots were snap-frozen in liquid nitrogen and stored at -80°C.

Per participant, one aliquot of serum per time-point was thawed and combined with 2DE lysis buffer [8 M urea; 2 M thiourea; 4% (w/v) CHAPS] at a ratio of 1:7, supplemented with inhibitor cocktail as detailed above. Total protein solubilization was carried out over 2 h at 4°C with intermittent (15–30 min) gentle vortexing and centrifugation at 500 × *g*. Solubilized serum samples were aliquoted and snap frozen, with 10 μl of each reserved for estimation of total protein concentration.

### Protein Concentration Estimation

Estimation of total protein concentration was carried out using a solid-phase protein assay as previously described (Noaman and Coorssen, 2018). Briefly, solubilized serum samples were serially diluted to yield concentrations appropriate for measurement against a linear calibration curve (0.5–0.05 mg/ml of BSA in 2DE lysis buffer). 1 μl of each dilution was dot-blotted in triplicate onto Whatman^TM^ 3MM chromatography paper (GE Healthcare, Chicago, IL, United States). Dried blots were washed with methanol for 5 min, dried under ambient conditions, and stained for 10 min with colloidal CBB (cCBB). Destaining was carried out for 5 min × 5 min with ddH_2_O, and blots were dried prior to imaging via reflective densitometry using the GS-900 Calibrated Densitometer (Bio-Rad, Hercules, CA, United States) followed by quantitation using ImageLab (Bio-Rad, Hercules, CA, United States) and Microsoft Excel 2013.

### Two-Dimensional Gel Electrophoresis (2DE)

Samples were assigned a numerical code and the order of sample analysis was determined with the use of a random number generator. Whole-serum 2DE was carried out as described previously (D’Silva et al., 2018). Briefly, 500 μg total serum protein was combined with rehydration buffer [2DE lysis buffer with 0.75% (v/v) pH 3–10 and 0.25% (v/v) pH 4–6 carrier ampholytes (Bio-Rad, Hercules CA, United States)], supplemented with TBP/DTT in the 1st hour, and acrylamide in the 2nd hour, for protein reduction and alkylation, respectively. 17 cm pH 3–10 non-linear IPGs were passively rehydrated for 16 h prior to isoelectric focussing (IEF), carried out at 17°C and 10,000 V for 75000 VH using a Protean i12 IEF cell (Bio-Rad, St. Louis, MO, United States), with multiple electrode wick changes during voltage ramping to facilitate desalting. Following IEF and prior to SDS-PAGE, IPGs were incubated for 20 min in equilibration solution {6 M urea, 0.375 M tris [pH 8.8), 2% (w/v) SDS, 10% (w/v) glycerol}, supplemented with 2% (w/v) DTT in the first 10 min followed by 350 mM acrylamide in the last, for reduction and alkylation, respectively.

SDS-PAGE was in hand-cast large-format (18 cm × 18 cm × 0.1 cm) 7–20%T gradient gels {0.375 M Tris [pH 8.8]; 0.1% (w/v) SDS; 0.1% (w/v) LDS; 0.05% (w/v) APS; 0.05% (v/v) TEMED (D’Silva et al., 2017)}, poured using a gradient former and multi-caster produced by the Brock University Machine Shop (such equipment may be purchased commercially). Electrophoresis was carried out at 4°C and 300 V for 15 min followed by 120 V until completion, typically for 24–26 h, using the Protean II XL system (Bio-Rad, Hercules, CA, United States). Resolved proteins were fixed in-gel with 10% (v/v) methanol, 7% (v/v) acetic acid for a minimum of 1 h, washed 3 min × 20 min with ddH_2_O, and stained for 20 h with cCBB followed by 5 min × 15 min destaining with 0.5 M NaCl (Noaman et al., 2017). Gels were imaged via transmissive densitometry using the GS-900 at highest scanning resolution (36.3 μm). For each of the five serum samples from each of the six participants, three 2DE replicates (technical *n* = 3) were resolved to ensure reproducibility.

### Image Analysis

Quantitative 2DE gel image analysis was done using Delta2D (DECODON Gmbh v4.7, Greifswald, Germany). Gel images were grouped into (A) pre-exercise, (B) 5 min post-exercise, and (C) 1 h post-exercise, as well as visit 2 (D) pre-muffin and (E) 1 h post-muffin. Images were meticulously warped, and a consensus gel image was created using ‘union fusion’ and automatically established detection parameters (average spot size, local background region in pixels, and sensitivity). From this image, a protein spot pattern was generated, manually edited to exclude artifacts, and transferred to all individual gel images for ‘100% spot matching.’

The resulting quantitation table which displayed average normalized spot volumes for each spot across each condition was used to determine significant changes in protein abundances. Spots which were statistically different (*p*-value < 0.05) in normalized volume between pre-exercise and 5 min and/or 1 h post-exercise, and 5 min post-exercise to 1 h post-exercise, with a ratio ≥1.1 or ≤0.9 and a relative standard deviation ≤30%, were considered genuine changes, and thus candidates for analysis by LC-MS/MS.

Comparisons of visit 1 pre-exercise and visit 2 pre-muffin samples, as well as visit 2 pre- and post-muffin samples, were carried out to ensure significant changes following HIIE were not attributed to day-to-day variability and/or food consumption.

### In-Gel Digestion and LC-MS/MS

In-gel digestion was carried out essentially as described previously (Wright et al., 2014). Manually excised protein spots combined from multiple gels were equilibrated briefly in 100 mM ammonium bicarbonate, and destained with 50% (v/v) acetonitrile and 50 mM ammonium bicarbonate prior to dehydration with 100% (v/v) acetonitrile. In-gel tryptic digestion with 3 ng⋅μl^-1^ trypsin in ammonium bicarbonate was carried out for 30 min at 4°C followed by 12 h at 37°C. Peptide solutions were recovered in microcentrifuge tubes and dried in a speed vacuum. Samples were shipped in microcentrifuge tubes at ambient temperature for LC-MS/MS analysis.

The MS analysis was carried out using a Q-Exactive mass spectrometer (Thermo Scientific), using a top 10 data dependent acquisition method with automatic switching between MS and MS/MS. Full-scan MS mode (375–1600 m/z) was operated at a resolution of 70,000 with automatic gain control and a target of 1 × 10^6^ ions. Ions selected for MS/MS were subjected to the following parameters: resolution 17,500, target of 1 × 10^5^ ions, 1.5 m/z isolation window, normalized collision energy 27.0 V and dynamic exclusion 20.0 s. Source ionization parameters were as follows: spray voltage, 1.9 kV; capillary temperature, 280°C; and s-lens RF level 50.0.

LC-MS/MS results were searched using Proteome Discoverer (version 2.2, Thermo Scientific) against the SwissProt human database (49,070 entries) in which raw files were searched using the Sequest HT algorithm. Peptides produced by trypsin proteolysis with a maximum of two missed cleavages were matched using precursor and fragment mass tolerances of 10 ppm and 0.02 Da, respectively. Propanamide (C) selected as a fixed modification, and oxidation (M), deamidation (NQ) and acetyl (protein N-term) were chosen as variable modifications. Peptide spectrum matches (PSMs) were verified based on *q*-values set to 1% false discovery rate (FDR). This resulted in the identification of multiple proteoforms (defined as differences in pI and/or MW). In these instances, only those hits with high sequence coverage (i.e., ≥5%) and number of unique peptides (i.e., ≥2) were accepted as identified proteoforms (Table 1). All raw mass spectrometry data has been deposited and is publicly available: ftp://massive.ucsd.edu/MSV000083129/raw/.

**Table 1:** Ratios of spot volumes for 5 min and 1 h post-HIIE when compared to pre-exercise and the proteins identified within those spots by LC-MS/M.

Spot number	Response 5-min PE (ratio/*p*-value)	Response 1-h PE (ratio/*p*-value)	Gene	Protein identified	Accession number	Theoretical MW/pI^∗^ (kDa/pI)	Observed MW/pI (kDa/pI)	Score/PSM	Seq. Cov %	Number of peptides/unique peptides
1	↔	↑	*SERPINA3*	α-1- antichymotrypsin	P01011	47.6/5.5	63.3/4.6	14/9	13	5/5
	1.1	1.2								
	0.4	0.02								
2	↔	↑	*AHSG*	α-2-HS- glycoprotein	C9JV77	39.4/5.7	61.2/4.5	529/232	13	5/5
	1.1	1.2	*SERPINA1*	(fetuin-a)						
	0.08	0.02		α-1-antitrypsin	A0A024R6I7	46.7/5.6		67/44	21	10/10
			*KNG1*	Kininogen-1	P01042	71.9/6.8			7	4/4
3	↔	↑	*AHSG*	α-2-HS- glycoprotein	P02765	39.4/5.7	60.0/4.5	674/274	19	11/11
	1.1	1.2		(fetuin-a)						
	0.08	0.02		α-1-antitrypsin	A0A024R6I7					
			*SERPINA1*			46.7/5.6		117/77	25	13/13
4	↑	↑	*SPERINC1*	Antithrombin-III	P01008	52.6/6.7	59.8/4.5	58/52	29	9/9
	1.2	1.3								
	0.02	0.005								
5	↔	↑	*SERPINA1*	α-1-antitrypsin	A0A024R6I7	46.7/5.6	60.0/4.7	805/524	55	27/27
	1.1	1.2								
	0.2	0.02								
6	↔	↑	*SERPINA1*	α-1-antitrypsin	A0A024R6I7	46.7/5.6	58.8/4.9	1676/940	61	33/33
	1.0	1.1								
	0.4	0.02								
7	↔	↑	*GN*	Vitamin D binding protein	P02774	53.0/5.5	57.4/5.0	1194/650	46	28/4
	1.1	1.3								
	0.3	0.004			D6RF35					
8	↔	↓	*IGHA1*	Immunoglobulin	P01876	37.6/6.5	64.5/5.1	391/184	44	15/5
	0.8	0.7		heavy constant α 1	A0A0G2JMB2					
	0.2	0.008								
			*IGHV3OR16-9*	Immunoglobulin heavy variable 3/OR16-9 (non-functional)	A0A0B4J2B5	10.7/8.7		9.9/4	31	2/2
9	↔	↑	*TF*	Serotransferrin	P02787	77.0/7.1	72.3/6.4	1868/1087	78	89/89
	1.0	1.2	*IGHM*	Immunoglobulin heavy constant μ	A0A1B0GUU9	51.9/5.8		101/63	35	17/17
	0.4	0.02								
			*HRG*	Histidine-rich glycoprotein	P04196	59.5/7.5		31/21	18	8/8
10	↑	↑	*CLU*	Clusterin (APOJ)	P10909	52.5/6.3	60.1/58.5	165/105	24	12/12
	1.4	1.7								
	0.002	0.0008								
11	↑	↑	*CLU*	Clusterin (APOJ)	P10909	52.5/6.3	43.5/4.6	338/230	26	11/11
	1.6	1.5								
	0.0000	0.0003								
12	↑	↑	*CLU*	Clusterin (APOJ)	P10909	52.5/6.3	41.8/4.7	14/10	11	2/2
	1.2	1.3								
	0.004	0.01								
13	↑	↑	*APOE*	Apolipoprotein E	P02649	36.1/5.7	37.7/5.0	31/26	29	9/9
	1.4	1.6								
	0.007	0.0002								
14	↔	↑	*APOE*	Apolipoprotein E	P02649	36.1/5.7	36.6/5.1	68/74	34	10/10
	1.1	1.3								
	0.4	0.02								
15	↔	↑	*APOE*	Apolipoprotein E	P02649	36.1/5.7	35.8/5.2	1511/815	68	26/21
	1.1	1.2								
	0.1	0.02								
16	↑	↑	*JCHAIN*	Immunoglobulin J chain	P01591	18.1/5.2	27.3/4.4	26/24	11	3/3
	1.2	1.3								
	0.04	0.01								
17	↑	↑	*JCHAIN*	Immunoglobulin J chain	P01591	18.1/5.2	27.3/4.5	86/83	26	4/4
	1.3	1.3								
	0.02	0.007								
18	↑	↑	*APOA1*	Apolipoprotein A-1	P02647	30.8/5.8	25.2/5.0	4400/1835	86	52/52
	1.3	1.3								
	0.02	0.007								
19	↑	↔	*IGKC*	Immunoglobulin κ constant	P01834	11.8/6.5	29.0/6.2	393/178	86	9/9
	1.3	1.0								
	0.04	0.8								
			*IGLC2*	Immunoglobulin λ constant 2	P0DOY2	11.3/7.2		240/124	56	4/2
			*IGKV3-15*	Immunoglobulin κ variable 3-15	P01624	12.5/5.2		58/34	26	2/2
			*IGLL5*	Immunoglobulin λ-like polypeptide 5	A0A0B4J231	23.1/8.8		52/35	27	4/2
20	↓	↓	*IGKC*	Immunoglobulin κ constant	P01834	11.8/6.5	27.5/6.2	462/194	53	6/6
	0.8	0.8								
	0.006	0.0004								
			*IGKV3-20*	Immunoglobulin κ variable 3-20	P01619	12.5/4.9		150/71	41	3/2
			*IGLC2*	Immunoglobulin λ constant 2	P0DOY2	11.3/7.2		129/67	60	5/2
			*IGLL5*	Immunoglobulin λ-like polypeptide 5	A0A0B4J231	23.1/8.8		42/29	29	5/2


*Some of the spots contained more than one clearly identifiable protein; presented here are the best hits (i.e., highest coverage and peptide count). PE, post-exercise; MW, molecular weight; kDa, kilodaltons; PSM, peptide spectrum matches; ↔, ↑, and ↓, directional change from pre-exercise levels; ratio, either 5 min or 1 h post-exercise spot volume/pre-exercise spot volume. ^∗^Tabulated theoretical molecular masses and isoelectric points predominantly relate to intact precursors. Please refer to databases (e.g., UniProt and ExPASy ProtParam) for MW and pI of potential cleavage products and gene/splice isoforms, as well as common PTMs*.

## Results

Each of the six young, healthy, male participants had an initial blood draw before engaging in an HIIE testing phase. Mean VO_2max_ was 50.4 ± 1.0 ml/kg/min, and *W*_max_ was 391.8 ± 8.3 W, the latter within the 80th percentile for healthy men in the age category assessed (Wang et al., 2010), indicating that the participants had adequate endurance potential. During HIIE trials, mean heart rates were >90% of the participants predicted HR_max_ (94.1 ± 5.3%) and immediately following they all responded ≥19/20 on the Borg scale (Borg, 1998). This confirmed that the HIIE trial was indeed high-intensity and successful in exhausting the participants. Following HIIE trials, blood was collected 5 min and 1 h post-exercise for 2DE analysis.

**FIGURE 2:**
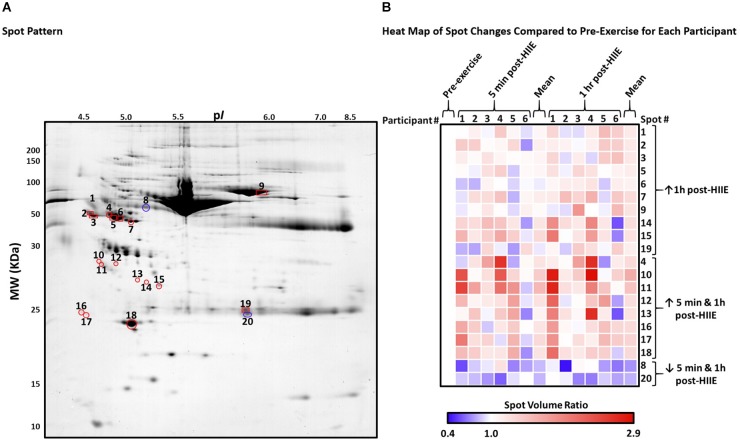
Spot pattern and fold changes for spots that changed significantly post-exercise and/or during recovery when compared to pre-exercise. **(A)** Representative gel image of the serum proteome, indicating the spots that changed significantly 5-min/1 h post-HIIE. Spots in red indicate a significant increase while blue indicates a decrease. Spots with a triangle indicate changes only 5 min post-HIIE, while squares indicate changes only during 1 h post-HIIE, and circles indicate changes both 5 min post-HIIE and 1 h post-HIIE when compared to pre-exercise. **(B)** Heat map of the changes for each participant for each spot; each cell is the average spot volume ratio change from 5 min and 1 h post-HIIE when compared to pre-exercise for each participant from the triplicate gels resolved for each participant. Blue and red indicate a decrease and increase, respectively, while white is no change when compared to pre-exercise levels. Since the ratios presented in the heat map were calculated based on the pre-exercise spot volume for each individual, pre-exercise then indicates no change; the first column (pre-exercise) is thus white to better visually emphasize the changes for each spot for each participant. Averages are also presented, which represent the mean of all participants and their replicates. Statistics (i.e., *p*-values and quantities) are presented in Table 1.

2DE enabled the resolution and detection of 977 consensus spots (i.e., spots which resolved consistently and were thus analyzed across all gels). Twenty spots were identified to have changed significantly from pre-exercise to 5 min post-exercise and/or 1 h post-exercise (*p* < 0.05; Figure 2A,B). Several high-quality/confidence database hits were returned following LC-MS/MS, identifying at least 15 different proteoforms within these spots (Table 1). These spot changes were not observed between visit 1 and 2 (pre-exercise, and pre-muffin consumption, respectively) samples, nor visit 2 pre- and post-muffin- consumption samples (not shown), indicating that neither day-to-day variability nor food consumption affected the proteoforms shown here to be associated with acute HIIE.

Table 1 summarizes the most confidently identified proteoforms within each spot analyzed which changed from pre- to 5 min and/or 1 h post-exercise. Additional peptide data from each spot can be found in Supplementary Data File S1. Average spot volume ratio changes for each participant are summarized in a heat map in Figure 2B, demonstrating the extents of biological variability.

Proteoforms that changed post-HIIE were largely from the protein families including serpins, apolipoproteins, fetuins, immunoglobulins, and albumins. The proteoforms that were found to have increased post-HIIE were fetuin-a, α-1-antitrypsin, vitamin D binding protein, histidine-rich glycoprotein, apolipoprotein J (clusterin), apolipoproteins E and A1, and immunoglobulin J chain; and those that decreased included immunoglobulin heavy constant α 1, immunoglobulin k constant, and β-2-glycoprotein 1. Apolipoprotein E, vitamin D binding protein, immunoglobulin J chain, and clusterin were each found in more than one fixed MW and pI gel region, suggesting resolution of different proteoforms (Jungblut et al., 2008; Thiede et al., 2013; Oliveira et al., 2014; Coorssen and Yergey, 2015; Zhan et al., 2018).

Of the spots changing in abundance, 8 of 20 were found to decrease in volume at 5 min post-HIIE and remained decreased 1 h post-HIIE. The proteoforms in these spots were identified as immunoglobulin heavy constant α 1 and immunoglobulin κ constant, respectively. The other 18 spots either increased only 1 h post-HIIE (α-1-antichymotrypsin, fetuin-a, α-1-antitrypsin, kininogen-1, serotransferrin, histidine-rich glycoprotein, and immunoglobulin κ constant) or at both 5 min and 1 h post-HIIE (clusterin, apolipoprotein E, immunoglobulin J chain, apolipoprotein A-1, and retinol binding protein 4).

**FIGURE 3:**
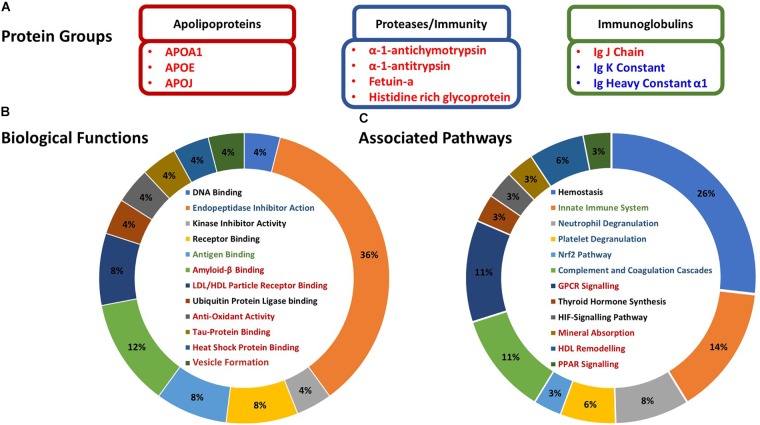
Characterization and associations of the proteoforms altered in response to HIIE. **(A)** Candidate exercise-regulated proteoforms that may be mediating the numerous multi-system health benefits we observe with exercise training. **(B)** Gene Ontology biological functions of the proteoforms altered post-exercise – identified with Uniprot. **(C)** Protein associated pathways of the proteoforms altered post-exercise – identified with PANTHER. Protein names in red indicate proteins that increased post-exercise, and proteins in blue indicate a decrease. Biological functions and associated pathways in red, blue, and green are related to apolipoproteins, proteases/immunity, and immunoglobulins, respectively.

Bioinformatic analyses suggested that most of the proteoforms which changed in abundance following HIIE were related to inflammatory responses, protein and lipid binding, antioxidant activity, metabolism and exosome formation (Figure 3A,B). Specifically, the apolipoproteins that increased post-HIIE (apolipoproteins A1, E, and clusterin) are associated with LDL/HDL particle receptor binding, amyloid β binding, tau protein binding, heat-shock protein binding and antioxidant activity; and the pathways they affect include GPCR signaling, mineral absorption, HDL remodeling, and PPAR signaling. Protease inhibitors and binding proteins that increased in abundance following HIIE (α-1-antichymotrypsin and α-1-trypsin and fetuin-a, respectively) were associated with amyloid-β binding (fetuin-a), and their pathways include neutrophil degranulation, platelet degranulation, Nrf2 pathways, complement and coagulation cascades, and mineralization (fetuin-a). Immunoglobulins affected by HIIE are associated with antigen binding and innate immunity (Figure 3B,C).

## Discussion

2DE coupled with LC-MS/MS enabled the resolution and identification of differentially abundant intact proteoforms, unlikely to have been specifically detected by a bottom-up MS-based ‘shotgun’ proteomic approach nor through targeted proteomic assays (e.g., western blotting, ELISA, and SOMAscan) since both methodologies generally assess only gross changes in abundances, with critical information pertaining to intact proteoforms and their unique physicochemical characteristics (that modulate function and localization) lost or unaccounted for. This is the first study to sequentially assess the human serum proteome following HIIE using a comprehensive top-down approach to identify significant changes in proteoforms (i.e., resolved protein species deviating in pI and/or MW from the theoretical values that are defined only on the basis of amino acid sequence; Table 1) (Jungblut et al., 2008; Thiede et al., 2013; Coorssen and Yergey, 2015; Zhan et al., 2018), implicating proteoforms associated with immune function, coagulation cascades, vitamins, protein and lipid metabolism, and proliferative and apoptotic pathways.

Many of these novel exercise-regulated proteoforms (i.e., exerkines) have been previously suggested as potential therapeutic targets in chronic diseases [i.e., Alzheimer’s disease (AD), type II diabetes mellitus and cardiovascular disease (CVD)] (Booth et al., 2012), supporting the notion that these proteoforms may mediate the longer-term health benefits associated with exercise training. These findings serve as initial evidence to further investigate the utility of these proteoforms as biomarkers for long-term positive health outcomes, though perhaps also for acute exhaustive exercise. Herein, the discussion will focus on the identified proteoforms that are altered following acute HIIE and their potential impact on immune function and the subsequent health benefits; however, it is important to keep in mind that these protein species have roles in health and disease other than those discussed here.

### HIIE Modulates Serum Proteins Known to Regulate Immune Function

It has been suggested that acute bouts of exercise may lead to a state of immune suppression which increases the risk of opportunistic infection (Peters and Bateman, 1983; Nieman et al., 1990; Gleeson, 2007). In contrast, others propose that immunity is improved as immune cells begin to localize in peripheral tissues (e.g., lungs and bone marrow) to increase surveillance and remove damaged/malignant cells (Matthews et al., 2002; Dhabhar, 2014; Campbell and Turner, 2018). Changes in proteoform abundance may be representative of varying post-translational modifications (PTMs) (Jungblut et al., 2008; Thiede et al., 2013), changes in expression, secretion or rate of degradation, or indicate an increase in distribution to peripheral tissues. Thus, certain tissues may experience an increase in homing factors resulting in an increase in inflammatory cells/factors being recruited to the periphery when a decrease is measured in serum. This is important to consider when moving forward with a discussion of these initial results, as well as in considering the design of future studies.

These data indicate that proteoforms of immune-regulatory proteins either increase (α-1-antichymotrypsin, fetuin-a, α-1-antitrypsin, apolipoprotein A1, clusterin, and immunoglobulin J chain) or decrease (immunoglobulin heavy constant α 1 and immunoglobulin k constant) in abundance post-HIIE. The immunoglobulin J chain is critical for production of secretory antibodies, which suggests there may be an increase in the secretion and priming of IgA and IgM, which are the initial responders to a specific immunologic defense (i.e., mucosal defense) (Woof and Mestecky, 2015). On the other hand, the decrease in immunoglobulins k and α indicates either that B cells are suppressed during HIIE or that there is a redistribution of immunoglobulins to the periphery to increase surveillance during and immediately following HIIE. Exercise induces the mobilization of B cells into the circulation; however, naïve B cells increase more in number than effector B cells (Turner et al., 2016). Although B cell function was not assessed in this study, given that J chain increases and immunoglobulin α decreases in abundance, we speculate that there is likely a redistribution of immunoglobulin α. These findings are thus consistent with the suggestions that exercise may play an important role in preventing infection, and indicate a potential mechanism as to how acute and chronic exercise may modulate or ameliorate the effects of pro-inflammatory cytokine (e.g., TNF-α and IL-1β) production in response to infection (Kohut et al., 2009).

The increase in circulating protease inhibitors α-1-antichymotrypsin and α-1-antitrypsin and their pro-forms, and the binding protein fetuin-a, suggest a suppression of neutrophil, platelet and mast cell degranulation (Hercz, 1974; Lebreton et al., 1979). This may be a compensatory response to the observed HIIE-induced increase in circulating neutrophils (Neves et al., 2015). α-1-antichymotrypsin also acts as a negative regulator of pro-inflammatory cytokine production (e.g., TNF-α and IL-1β) and innate immunity, thus protecting against systemic inflammation (i.e., lethal endotoxemia and sepsis) (Wang and Sama, 2012). Furthermore, α-1-antitrypsin appears to have other anti-inflammatory properties (Bergin et al., 2012), which include protection against apoptosis induced by pro-inflammatory cytokines (e.g., TNF-α, IL-1B, and IFN-γ) (Kalis et al., 2010), inhibition of toll-like receptor 4 and 2 signaling (Jonigk et al., 2013), and regulation of neutrophil chemotaxis induced by soluble immune complexes (Bergin et al., 2010). Additionally, apolipoprotein A1 appears to be a critical anti-inflammatory mediator during the acute phase, and is known to inhibit pro-inflammatory cytokine production in monocyte-macrophages (e.g., TNF-α and IL-1β) (Hyka et al., 2001). Furthermore, in a model of non-alcoholic steatohepatitis, overexpression of clusterin has been shown to inhibit pro-inflammatory cytokine production (e.g., TNF-α and IL-1β) and hepatic macrophage infiltration, and to abolish hepatic fibrogenesis through the activation of the transcription factor Nrf2 which activates the antioxidant response element (Park et al., 2018), thus increasing proteins that lead to detoxification and elimination of reactive oxygen species and electrophilic agents (Falgarone and Chiocchia, 2009; Nguyen et al., 2009).

Other identified proteoforms, including histidine-rich glycoprotein (Poon et al., 2011), transferrin (Mancini et al., 2016), antithrombin III (Inthorn et al., 1998), kininogen I (Bryant and Shariat-Madar, 2009), and vitamin D binding protein (Gomme and Bertolini, 2004), have also been shown to have either anti-inflammatory properties or regulate the immune response. Taken together, these post-HIIE molecular responses suggest an overarching anti-inflammatory response rather than immune suppression, which is often said to occur following intense exercise. While these proteoforms are not the classical inflammatory cytokines used to describe systemic inflammation, many have been extensively shown to have anti-inflammatory effects. We have previously shown that pro-inflammatory cytokines, TNF-α and IL-1β, transiently increase post-HIIE and return to baseline 1 h post-HIIE (Mezil et al., 2015). The increase in α-1-antichymotrypsin, α-1-antitrypsin, apolipoprotein A1, and clusterin shown here, may indicate a protective mechanism through anti-inflammatory and antioxidant activity, which is sustained through recovery (i.e., 1 h post-HIIE).

One of the major underpinnings of chronic disease is inflammation caused by obesity or a sedentary lifestyle. There is an overt variation in immune cells as well as platelets, and molecular makeup within the circulation and in peripheral tissues in individuals with chronic disease (i.e., metabolic syndrome). The data here thus indicate that there is a shift toward the regulation of these inflammatory responses/pathways through an increase in anti-inflammatory agents. Thus, the modulation of these immune-regulatory proteins post-HIIE may have a role in the prevention and treatment of many chronic diseases.

### HIIE Modulates Proteoforms That May Mediate the Prevention of Several Chronic Diseases

Exercise is the ideal first option therapy for prevention and treatment of numerous chronic diseases (e.g., accelerated aging, metabolic syndrome, Type II diabetes mellitus, and CVD) (Booth et al., 2012). Specifically, fetuin-a has been shown to have cytoprotective activity against oxidative injury in neuronal cells (Kanno et al., 2017) and inhibits calcification of atherosclerotic plaques in patients with type II diabetes mellitus (Emoto et al., 2010). The anti-inflammatory effects of fetuin-a and α-1-antitrypsin, as well as their potential roles in mediating insulin sensitivity in response to exercise training (Kalis et al., 2010; Blumenthal et al., 2017), may thus be an avenue of interest in efforts to find therapeutics that act as mimetics for exercise and thus combat metabolic diseases/insulin resistance. In this regard, however, it is important to note that α-1-antitrypsin was identified in five different resolved spots, and fetuin-a in three, each of which differed markedly in MW and pI from the theoretical values. Therefore, these effects are likely mediated (perhaps selectively) by various proteoforms of the canonical species, emphasizing the importance of clearly identifying active species prior to the development of therapeutics. Focussing on amino acid sequences alone is insufficient in terms of identifying effective biomarkers and designing new therapeutics.

Lipid handling/storage appears to have an important role in the inflammatory response in individuals with atherosclerosis, CVD, and neurodegenerative diseases (Libby et al., 2013). Apolipoproteins are a major class of lipid binding proteins that are thought to be associated with several chronic diseases, given their roles in binding and clearing various lipids (i.e., cholesterol), forming HDL (apolipoprotein A1), and degrading LDL (Horejsi and Ceska, 2000). Cholesterol accumulation contributes to the activation of the immune response and subsequent increase in pro-inflammatory cytokines, which can lead to pathological chronic inflammation (Libby et al., 2013). Thus, the increase in abundance of apolipoproteins following HIIE, which regulate the handling and clearance of cholesterol and promote an increased HDL:LDL phenotype, may be a means of preventing systemic inflammation and adequately regulating lipoprotein abundance/ratios.

Clusterin protects cardiomyocytes from apoptosis through the Akt/GSK-3β signaling pathway (Jun et al., 2011), and protects the heart from damage caused by myocardial infarction (Foglio et al., 2015), transplant (Li et al., 2011), or myocarditis (McLaughlin et al., 2000) (reviewed in: Pereira et al., 2018). It is thought that apoptosis of cardiomyocytes is one of the main age-related contributors to development of heart disease (Kumar et al., 2002; Goldspink et al., 2003), thus the increase in abundances of three clusterin proteoforms following acute-exercise may be responsible for the lower levels of heart disease seen in individuals who are more active (Buchner, 2009). Clusterin appears to be associated with AD risk (Schrijvers et al., 2011); however, it is most likely a compensatory/neuroprotective response (reviewed in: Nuutinen et al., 2009) as it is associated with AD severity, but not with the risk of developing AD at follow up. Interestingly, clusterin has been shown to inhibit amyloid formation through (1) binding amyloid-β or enhancing its clearance across the blood–brain barrier (Yerbury et al., 2007); (2) clearance by endocytosis of amyloid-β aggregates and cell debris by brain phagocytes (Bell et al., 2007); and (3) inhibition of complement activation (Nuutinen et al., 2009). Taken together, these findings suggest clusterin is neuroprotective rather than complicit in AD progression and is most likely a component of a compensatory response in AD.

Exercise also has neuroprotective effects through the stabilization of apolipoprotein E (Soto et al., 2015), and is integral for maintaining blood–brain barrier integrity (reviewed in: Montagne et al., 2017). Multiple sclerosis is a disease that is characterized in-part by inflammation which provokes the progression and pathogenesis of the disease. Exercise training has been shown to moderate symptoms and disease progression, thereby improving the quality of life of individuals with multiple sclerosis (Stuifbergen et al., 2006). Apolipoproteins E and A play critical roles in cholesterol homeostasis and subsequent anti-inflammatory actions, as well as having a potential role in clearing amyloid-β from the brain into the circulation (Robert et al., 2017). These functions are critical for neuronal health and regeneration, indicating potential mechanisms for how exercise attenuates the progression and improves the quality of life of patients with multiple sclerosis (reviewed in: Gardner and Levin, 2015) and other neurodegenerative diseases. Apolipoproteins E and A1 also appear to promote the regression of atherosclerosis in diet-induced hypercholesterolemia and advanced aortic atherosclerotic lesions (Raffai et al., 2005), and appear to be critical in the regulation of lipid profiles (prevention of hyperlipidemia) and subsequent inflammation-induced atherosclerosis (Centa et al., 2018; Favari et al., 2018). Apolipoprotein E also appears to be a significant component of extracellular vesicles (e.g., exosomes, ectosomes) and may have a role in their formation (Looze et al., 2009; Nikitidou et al., 2017), which could indicate a novel mechanism for increased clearance of amyloid-β. Apolipoprotein A1 has been suggested to be an emerging risk biomarker for CVD (Florvall et al., 2006; Upadhyay, 2015), is a critical component of HDL, and appears to be a key component in the inhibition of atherosclerotic plaque formation (Favari et al., 2018). Taken together, there appear to be several plausible mechanisms and targets that HIIE may work through to explain the role of exercise in the prevention of metabolic syndrome, CVD, and neurodegenerative diseases.

Despite our efforts to control experimental variables there are some limitations to this study, which include (1) a relatively small sample size (*n* = 6); (2) only two time-points post-exercise, which did not allow us to determine if or when the identified proteoforms returned to baseline (or whether there are critical later changes); and (3) relatively limited in-gel protein detection sensitivity [∼2 ng/ml or ∼0.06 ng of total protein in a spot with cCBB staining (Noaman et al., 2017)], which, while rivaling standard shotgun approaches for peptides, is less than claims made for targeted albeit likely less selective proteomic techniques (e.g., ELISA). Future studies will focus on validating the species identified here as post-exercise biomarkers. Importantly, the characterization of the acute response before and after exercise training will aid in understanding the (mal)adaptation events that occur with these proteoforms. Thus, repeating the study with more time-points following acute exercise (recovery) and looking at the effect of exercise training may reveal if, and when these exercise-regulated proteoforms (exerkines) return to baseline. Additionally, assessing other sub-fractions of whole blood in this way will increase the detection sensitivity of lower abundance proteoforms (e.g., microvesicles) (Whitham et al., 2018), allow for the detection of proteoforms that may be lost during clotting (i.e., serum vs. plasma) (Kim et al., 2007), provide further understanding of the anti-inflammatory effects of exercise (e.g., peripheral blood mononuclear cells), and elucidate the tissues/cell types of origin and assess which tissues are affected by these exercise regulated proteoforms (i.e., tissue cross talk).

Overall, it is noteworthy that few of the proteins identified closely matched their theoretical pI and MW (i.e., did not conform to identification based *only* on amino acid sequence; a major shortcoming of relying purely on existing databases) and that ∼50% were found in more than one spot, clearly emphasizing the fundamental importance of proteoforms in biological mechanisms and thus the critical importance of using analytical approaches that can resolve these, as they represent specific targets for the development of future biomarkers and therapeutics.

## Conclusion

The 2DE-based top-down approach used here to assess the serum proteome response to HIIE revealed significant perturbations in the abundance of proteoforms, with roles in regulating immune function in health and disease during the minutes and hour following exercise, providing new insights into the mechanism(s) underlying how exercise may exert some of its numerous associated health benefits. We propose that these potential serum biomarkers be further validated for their utility in assessing recovery, exertion, and health status as well their importance in the anti-inflammatory mechanisms modulated by exercise.

## Data Availability

The datasets generated for this study can be found in Mass Spectrometry Interactive Virtual Environment (MassIVE), ftp://massive.ucsd.edu/MSV000083129/raw/.

## Author Contributions

NK, NN, JC, and PK conceived and designed the study. SC, JC, and PK obtained funding. NK and NN collected the samples. NK, NN, MP, and SC performed the experiments. All authors contributed to data analysis and interpretation of the results. NK and NN drafted the manuscript and did the subsequent revisions. All authors edited and revised the manuscript and approved the final version submitted for publication.

## Conflict of Interest Statement

The authors declare that the research was conducted in the absence of any commercial or financial relationships that could be construed as a potential conflict of interest.
